# Detection, Verification and Analysis of Micro Surface Defects in Steel Filament Using Eddy Current Principles, Scanning Electron Microscopy and Energy-Dispersive Spectroscopy

**DOI:** 10.3390/s23218873

**Published:** 2023-10-31

**Authors:** Kim Sang Tran, Bijan Shirinzadeh, Armin Ehrampoosh, Pan Zhao, Yaoyao Shi

**Affiliations:** 1 Robotics and Mechatronics Research Laboratory (RMRL), Department of Mechanical and Aerospace Engineering, Monash University, Melbourne, VIC 3800, Australia; kim.tran4@monash.edu (K.S.T.); armin.ehrampoosh1@monash.edu (A.E.); 2 The Key Laboratory of Contemporary Design and Integrated Manufacturing Technology, Ministry of Education, Northwestern Polytechnical University, Xi’an 710072, China; pan.zhao@nwpu.edu.cn (P.Z.); shiyy@nwpu.edu.cn (Y.S.)

**Keywords:** eddy current, steel filament, surface defect, longitudinal scratch, inclusion

## Abstract

In the current industrial revolution, advanced technologies and methods can be effectively utilized for the detection and verification of defects in high-speed steel filament production. This paper introduces an innovative methodology for the precise detection and verification of micro surface defects found in steel filaments through the application of the Eddy current principle. Permanent magnets are employed to generate a magnetic field with a high frequency surrounding a coil of sensors positioned at the filament’s output end. The sensor’s capacity to detect defects is validated through a meticulous rewinding process, followed by a thorough analysis involving scanning electron microscopy (SEM) and energy-dispersive spectroscopy (EDS). Artificial defects were intentionally introduced into a sample, and their amplitudes were monitored to establish a threshold value. The amplitude signal of these created defect was identified at approximately 10% FSH, which corresponds to a crack depth of about 20 µm. In the experimental production of 182 samples covering 38 km, the defect ratio was notably high, standing at 26.37%. These defects appeared randomly along the length of the samples. The verification results underscore the exceptional precision achieved in the detection of micro surface defects within steel filaments. These defects were primarily characterized by longitudinal scratches and inclusions containing physical tungsten carbide.

## 1. Introduction

Non-destructive testing (NDT) is by far the most important component of modern inspection technology, and it contributes an important role in ensuring that objects have reliable performance in its shelf-life. Especially, the application of NDT in manufacturing industries, such as for steel wire, is an integral element of the state-of-the-art inspection. Steel wire is used as a reinforcement material in the construction of tire belts, beads, and sidewalls, which are responsible for providing strength and stability to the tire [1,2]. It is well known that over 200 different materials are used in the production of tires, including high-tensile-strength steel filaments [3] and the application of NDT in this field is really necessary. Since micro surface defects are very small and cannot be seen with our eyes or any other normal inspection method.

**Figure 1:**
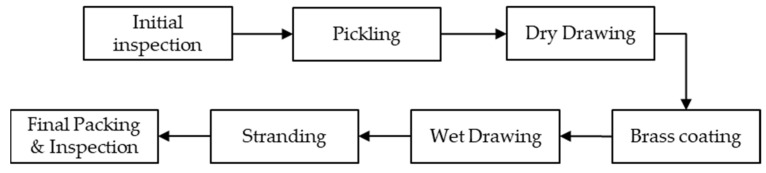
Steel filament manufacturing procedure.

The production of steel wire begins with a pickling process in which the coils undergo cleaning using hydrochloric acid and water to remove rust after initial inspection. The wire rods are then drawn into thinner diameters through multi-dies while in a dry condition using a special lubricant in powder form. The wire is then coated with a layer of brass, comprising copper and zinc. These brassed wires are then transferred to the wet-drawing process in which they are subjected to multi-pass drawing using a wet lubricant. Finally, the wires are pulled into spools at the end of the wet drawing process. The individual steel wires are then stranded together to form a steel cord, as shown in Figure 1.

In the steel wire manufacturing industry, one of the major challenges for steel wire suppliers is to identify failure modes in micro/nano scale due to their influence. With the occurrence of micro surface defects, the risk of steel wire breakage steadily increases, which can lead to serious accidents. Identifying and addressing these defects is crucial to ensure the safety and reliability of steel wire products. Many solutions have been suggested to minimize the likelihood of wire breakage in manufacturing. However, various technical or quality issues remain to be resolved in production workshops [4,5]. Therefore, the utilization of cutting-edge inspection technology is essential for quality control. 

Over the decades, the demand for visual inspection during the manufacturing process has significantly increased. In particular, non-destructive testing (NDT) has been widely used as a quality control gate to ensure that defective products are not delivered to end-users. Due to the high cost of manual inspection, automated observation system for quality control is preferred to replace human labor, and to improve overall equipment effectiveness (OEE) and productivity for manufacturers [6]. The utilization of modern techniques or prediction methodologies in human-controlled device can improve cycle time while also reducing production costs and waste [7,8,9,10].

As a part of the NDT industry, the eddy current principle finds extensive use in various applications, including automotive, aerospace and steel inspections. In terms of micro surface defect inspection, conventional monitoring methodologies have demonstrated limited responsiveness, making it applicable only in small scales, with high costs and very poor productivity [11]. 

A non-destructive testing approach was established by combining mechanical and magneto techniques, enabling the identification of signals emanating from surface defects on the wire [12]. To accurately determine the positions of defects on the goods, the utilization of sensor techniques or robot algorithms can be beneficial. These approaches can help in precisely localizing the defective objects within the product [13]. Another type of sensor used to detect defects on wire ropes is based on a signal called magnetic flux leakage. The concept was developed using the orthogonal test method [14].

The fatigue characteristic of the wire was significantly impacted by the presence of micro-defects when wires used. In a test conducted using wire rod specimens with surface defects, the behavior of steel wire was analyzed in terms of fatigue, where the presence of micro surface defects caused early wire breakage [15]. Other research efforts focused on the causes of fatigue in wire materials. In these studies, the role of micro surface defects was investigated, and the origin of fatigue cracks was found to be tiny surface defects in the initial phase of the materials [16]. Finite element simulation was used to predict the origin of failure in metal and carbon-fiber composite materials [17,18,19].

Visual inspection is essential in the cutting-edge manufacturing environment, particularly in aerospace, automotive, and three-dimensional printer engineering. A computer program was installed around the hot-rolling process using a multi-camera system to detect surface defects on wire rods. Another approach called WR-YOLO was also presented to detect surface damage in steel wire ropes. The equipment included a camera and a computer to monitor the broken wire situation on its surface during the stranding process, However, the system was too complicated to install and was not feasible for mass production with various wire diameters. [20,21]

The grooves in steel wire were analyzed to determine the impact of groove width and depth on wire quality. The results of the experiments conducted during production using the multi-die drawing process were compared to those simulated using finite element analysis [22]. In non-contact techniques applied in the steelmaking industry, ultrasonic reverberation spectroscopy was used to detect failures in steel wires. Various experiments were conducted under a magnetic field to enable signal detection to pinpoint the location of the failures [23]. Furthermore, a survey was performed to find defects in the fabric, however it was challenging to explore owing to the extensive stochastic variation, stretching, and distortion of fabric defects influenced by environment factors [24]. 

Another textile inspection method called computer vision was used to demonstrate the appearance of various typical defects by a fabric monitoring system [25,26]. Spectral approaches were also used in this field, but their issue was the accurate localization [27,28,29]. Micro surface defect detection on the surface of wire rods was the target of such image technology. This approach detected the position of failure; however, when using these image technologies, figuring out the defects accurately is not straightforward owing to the scale of the objects and the vignetting background [30].

In addition to the detection, surface defect analysis must also be considered. Various methodologies are based on signal processing and are not able to give a detail in defect analysis because they are not able to collect the specimen containing the surface defects. 

In the state-of-the-art industry, steel makers have the responsibility to reduce the risk of delivering faulty goods to the market. To achieve this goal, several inspection approaches using cutting-edge technologies have been developed to enhance product quality, overall equipment effectiveness, cycle time, etc. Among the various inspection technologies, vision-based monitoring is a common approach used to detect defects in different steel wire [31,32,33]. However, image technology alone is insufficient for the efficient examination or prevention of unacceptable products from being sent to the market given the large production volume and the diverse range of defects’ shapes on the surface, as opposed to the limited shapes of reference samples. Moreover, the steel wire manufacturing process operates at high speeds (input 400 m/minute) and high temperatures (over 300 °C) to produce steel filaments with very small diameters—under 0.5 mm.

This paper introduces an innovative methodology for the early identification of micro defects on 0.38 mm steel filaments. It intends to provide the approach to inspect, verify and analyze micro surface defects in steel filaments that can accurately optimize high-speed manufacturing processes and improve the quality of products. The proposed methodology utilizes the eddy current principle to address technical challenges related to enhancing quality and to tackle the problem of micro surface defects in the steel-cord-manufacturing industry. 

The remaining sections of this paper are structured as follows: The research methodology is provided in Section 2. Experiment setups are mentioned in Section 3. The experimental results are discussed in Section 4, and conclusions are given in Section 5.

## 2. Research Methodology

### 2.1. Eddy Current Principle

**Figure 2:**
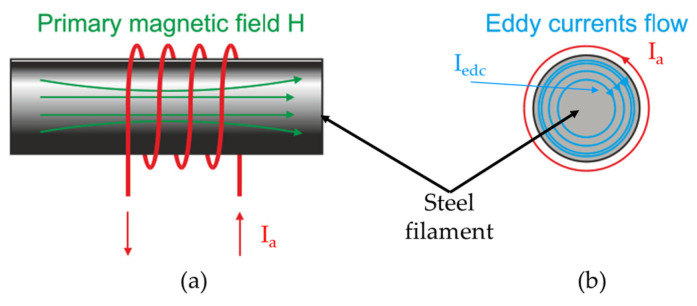
(**a**) Encircling coil with steel filament; (**b**) eddy current induced around steel filament.

The Eddy current is established based on the principle of electromagnetism as the basic for carrying out experiments [34]. An encircling test coil was utilized to monitor the micro surface defects of the steel wire during the wet-drawing process. The electrical inspection signals are generated by the electromagnetic interaction between the test coil and steel filament when this filament passed through the coil. When the test coil is activated by an alternating current $I_{a}$, it produces an alternating magnetic field H, in turn inducing an eddy current $I_{edc}$ in the steel filament, as shown in Figure 2 [9].

One of the key parameters of the sensor coil is impedance *Z*_0_, which is a complex number, as described in Equation (1):

(1) $$Z_{0}=R_{0}+jX_{0}$$

where $R_{0}$ is the real part, and $X_{0}$ stands for the imaginary component of impedance $Z_{0}$.

Since steel wire goes through the test coil, eddy currents appear on the steel wire, leading to a secondary field that is opposite with the primary field. Hence, another impedance is created, which is defined in Equation (2) [9]:

(2) $$Z_{c}=R_{c}+jX_{c}$$

where $R_{c}$ is the real part, $X_{c}$ stands for the imaginary part of new impedance, $X_{c}=j{\pi fL}_{c}$, and f and $L_{c}$ are frequency and induction coefficient, respectively.

### 2.2. Standard Penetration Depth and Defect’s Depth

Frequency is a part of the testing sensor coil, and when the applied frequency increases, the inductive reactance of test coil will increase accordingly, as indicated in Equation (3) [9]:

(3) $$X_{0}=j2{\pi fL}_{0}$$

where f and $L_{0}$ are frequency in Hertz (Hz) and inductance coefficient in Henrys (H) of the coil, respectively.

The distribution of eddy current is non-uniform across the complete volume of test specimens. It is more concentrated near the surface and gradually decreases exponentially when the distance from the surface increases. The current flux pattern is described in Equation (4) [9]:

(4) $$\vec{J}=J(z,t)\times \vec{u}$$

where $\vec{u}$ represents the unitary vector, J(z,t) denotes the magnitude of current density as a function of depth z and time t. The mathematical representation of the current density along the depth is presented in Equation (5) [35]:

(5) $$J(z)=J_{0,max}e^{-\frac{z}{\delta }}e^{j(\alpha_{0}-\frac{z}{\delta })}$$

where $J_{0,max}$ represents the maximum current density on the surface, and the standard penetration depth δ refers to the depth where the eddy current density reduces to approximately 37% compared to its surface level value. The parameter $\alpha_{0}$ indicates the phase at t=0 and z=0 and the ratio between z and δ stands for the phase lag. Equation (6) indicates the real part of the current density, and the phase of the current density changes by 1 radian as the distance traveled from the surface is δ [35]:

(6) $$J(z,t)=Real(J{\left(z\right)e}^{j\omega t}=J_{0,max}e^{-\frac{z}{\delta }}cos(\omega t+\alpha_{0}-\frac{z}{\delta })$$

The standard penetration depth δ is expressed by Equation (7), and it is influenced by the electrical conductivity σ, the magnetic permeability of the monitored specimen μ, and the applied frequency f, ω=2πf.

(7) $$\delta =\sqrt{\frac{2}{\sigma \mu \omega }}$$

In this experiment, the maximum frequency of 1000 kHz was chosen, $\sigma =\frac{1}{\rho },\;\rho =1.43\times 10^{-7}$ Ohm.m, and $\mu =1.26\times 10^{-4}\;H/m$ [36,37]. Therefore, the standard penetration depth is identified as 19.01 μm, which satisfies the condition that the thickness of the steel filament should be greater than or equal to 3δ to ensure that the sample thickness alterations do not impact the measurements. Because the concentration of eddy currents is higher on the surface and diminishes as one moves deeper into the steel filament, a phenomenon commonly referred to as the skin effect occurs when the depth at which the eddy current density decreases to $\frac{1}{e}$, i.e., approximately 37% of the surface density. At a depth of 2δ, the eddy current density has reduced to ${\left(\frac{1}{e}\right)}^{2}$ or 13.5% of the surface density. By 3δ, the eddy current density decreases to a mere 5% of the surface density [9].

## 3. Experiment

### 3.1. Sensor Coil Configuration

**Figure 3:**
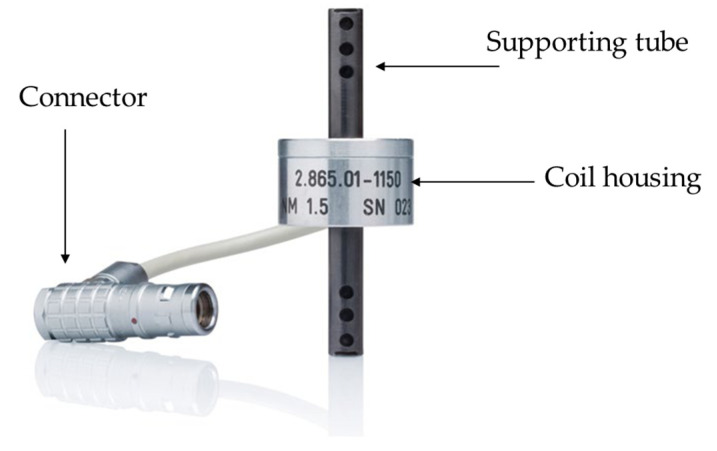
Encircling sensor coil.

A Defectomini sensor system from Foerster was used in the experiment. The test coil (sensor type 2.865.01-1050) consists of a coil housing with supporting tube on each side and the centrally positioned coil housing stores the coil unit, as illustrated in Figure 3. The connector is connected to the pre-amplifier, which is linked to the instrument (Defectomat CI). 

### 3.2. Experimental Setup

**Figure 4:**
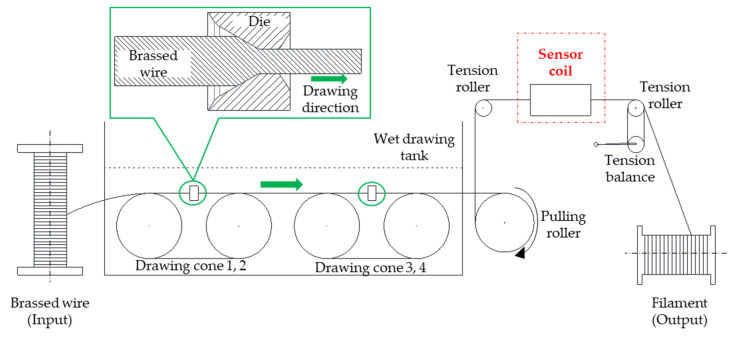
Wet-drawing process and sensor coil position.

A set of encircling sensors was placed on the top of wet-drawing machine, as shown in Figure 4, where the filament went through the sensor during the drawing process. The input material for the drawing process was steel wire of 1.90 mm diameter, which passed through the drawing machine, where it was reduced in diameter via a multi-die system in the wet lubricant tank. The output of the drawing process was the filament, which is inspected for quality using the eddy current sensor. 

**Figure 5:**
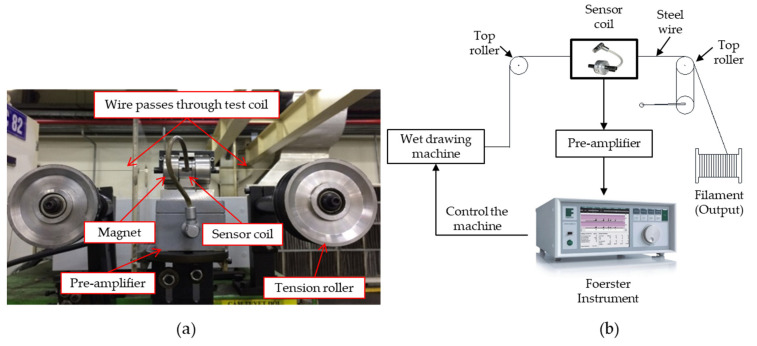
(**a**) Sensor installation on a wet-drawing machine; (**b**) experimental setup diagram.

The sensor coil was situated between two tension rollers at the machine’s output end, which was precisely aligned with the wire path. It was linked to a pre-amplifier, and the resulting signal was subsequently directed to the instrument, as depicted in Figure 5. This instrument was employed to define the criteria for a non-defective product. 

### 3.3. Establish Working Condition

**Figure 6:**
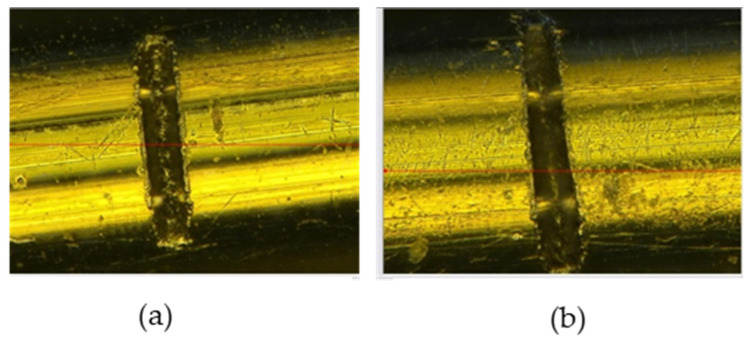
Artificial cracks under scanning electron microscope (SEM). (**a**) Crack’s depth of 20 µm; (**b**) crack’s depth of 80 µm.

Table 1 and Figure 6 display the introduction of artificial cracks at depths of 20 and 80 µm onto the surface of a 0.38 mm filament sample, which were measured using the reference red line in Figure 6.

**Figure 7:**
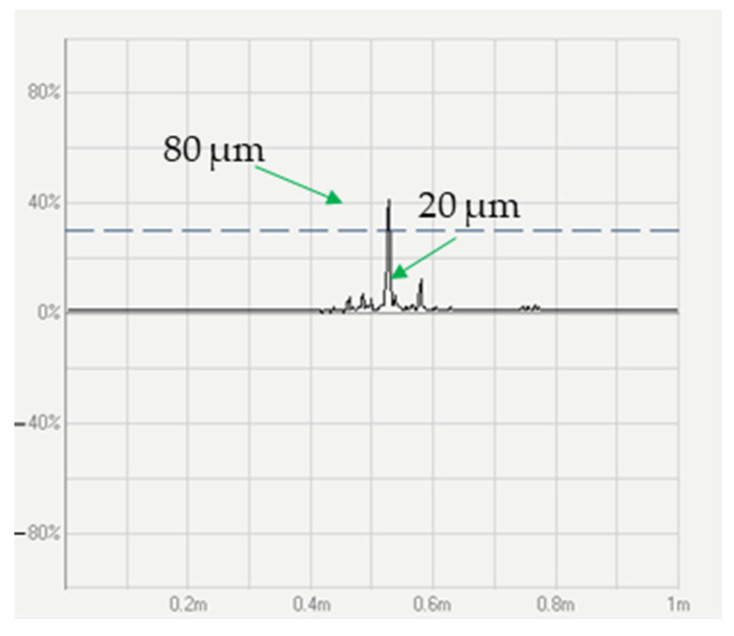
Amplitude signals of created cracks with the depth of 20 µm and 80 µm.

The specimen containing the created cracks was stretched and placed through the sensor in order to monitor the amplitude signal. The frequency was controlled at maximum level of 1000 kHz to identify the surface defects. Consequently, the amplitude signals of the introduced defects amounted to around 10% and 41% of the full-screen-height (FSH) amplitude for depths of 20 and 80 µm, respectively. Additionally, noise was also observed along with the defects, with a level of 3% attributed to the rusting of the wire, and the dotted line shows the reference threshold at 30% FSH, as illustrated in Figure 7.

If the threshold value is set at 10% FSH to identify the defects with a depth of 20 µm, the sensor will detect any defects containing cracks deeper than 20 µm during the experiment. Based on the testing results, the threshold of amplitude signal was set of 10% FSH to stop the machine when desired defect appears.

### 3.4. Detection of Surface Defects

Based on the established setting conditions from previous steps, the sensor was installed at the output stage of the drawing process. Non-defective specimens should have a length of 38 km without any defect or with minor defects characterized by an amplitude signal below 10%. When an amplitude signal exceeds the threshold value, the drawing process will be stopped. 

### 3.5. Verification of Surface Defects

To verify the detected defects on filaments, 13 defective samples were selected and pulled back through the sensor coil to verify the signals of micro surface defects and compared them to amplitude signals measured during the experiment.

When the sensor detects desired defects, the machine keeps running for an additional 26 m before stopping. Then, the output filament was manually pulled back through the sensor to capture the defective signal and compared with those obtained from the system. The defects were also identified under an optical microscope before cutting them into small pieces containing the surface defects, which were then analyzed by SEM and EDS.

## 4. Experimental Results

### 4.1. Detection of Micro Surface Defects

In this study, 182 specimens were tested on the steel-wire-manufacturing process, the sensor system identified 48 flawed samples, as outlined in Table 2. This translated to a defect ratio of 26.37%, indicating the proportion of products with surface defects exceeding a depth of 20 µm.

**Figure 8:**
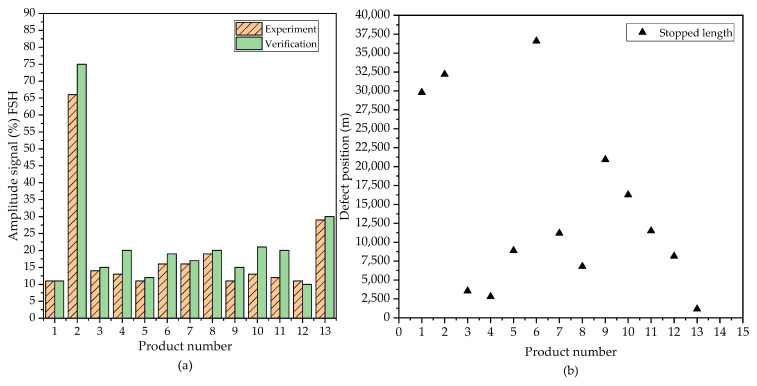
(**a**) Comparison of the amplitude signals for defects during production and verification; (**b**) Position of defects on unacceptable products.

Figure 8a illustrates the accurate detection of the identified flawed samples. The disparity was minimal, and the amplitude signals for most defects falls within the range of 10% to 35%, except for a single defect for which the amplitude signal significantly exceeded the others, with an average around 70%. 

Figure 8b illustrates the position of the micro surface defects where the sensor system stopped the machine upon detecting the defect exceeding a depth of 20 µm. Surface defects manifest randomly along the length of each sample.

### 4.2. Defects Analysis

**Figure 9:**
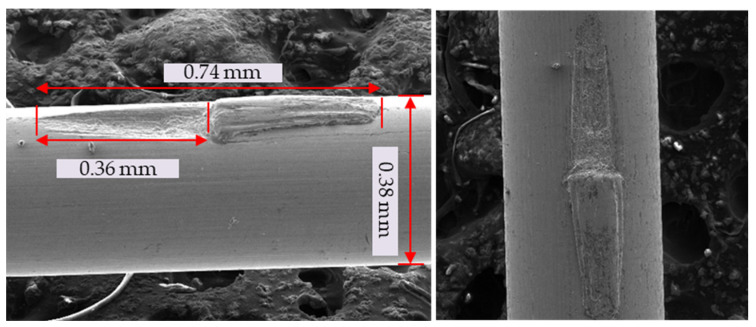
Surface defect in filaments imaged under SEM.

After detecting micro surface scratches, the machine ran for an additional 26 m before coming to a complete stop. By identifying the excess length and rewinding it backward using the sensor coil, the same signals were captured and compared with those recorded in the system for the previous specimen. Eventually, surface defects in steel filament were confirmed and clarified under a microscope, as indicated in Figure 9.

**Figure 10:**
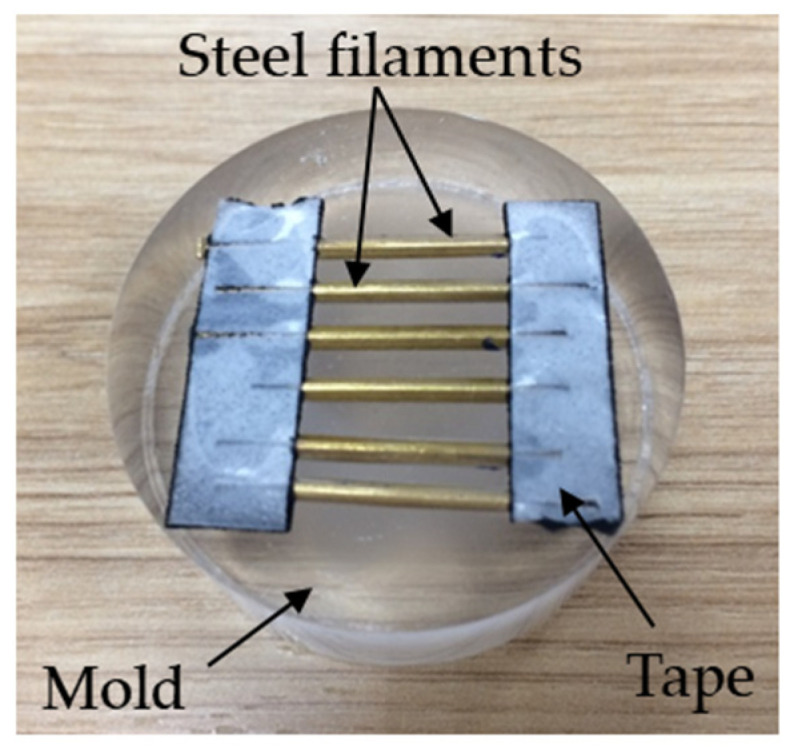
Steel filaments containing surface defects.

Under an optical microscope, the components containing the defect were sectioned into smaller fragments and affixed in parallel with tape prior being placed into a mold, as depicted in Figure 10.

**Figure 11:**
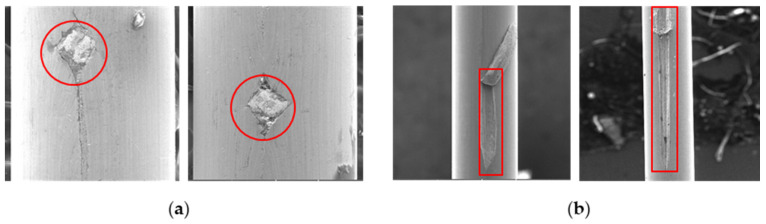
Typical types of surface defects shown in red frames: (**a**) inclusion defects; (**b**) scratch defects.

Figure 11a shows a unique defect containing tungsten carbide (WC) inside. On the other hand, Figure 11b illustrates the outcome of scratches, allowing for the prediction of the start and end points of such scratch defects. Most of these defects resembled the pattern shown in Figure 11b.

**Figure 12:**
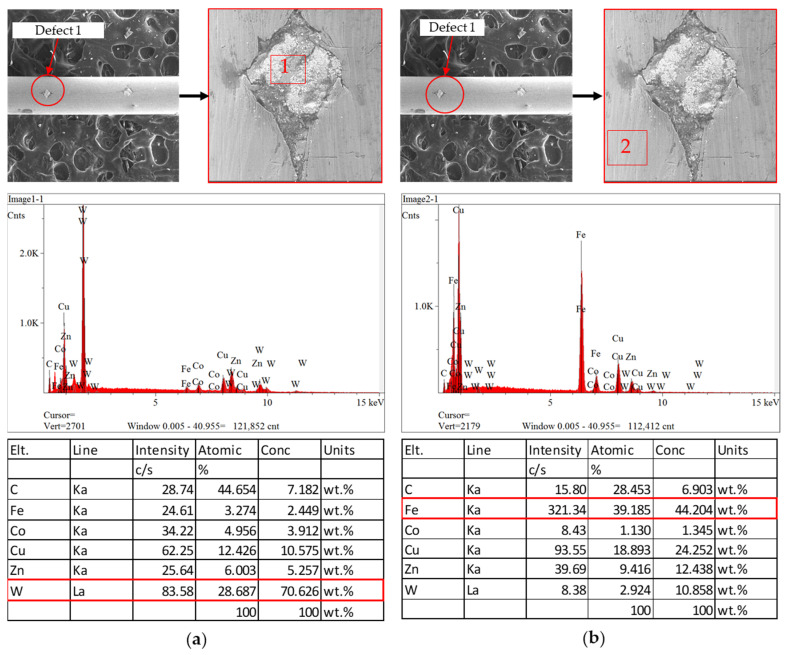
Results of EDS analysis for inclusion defect 1. (**a**) Inside of defect; (**b**) outside of defect.

**Figure 13:**
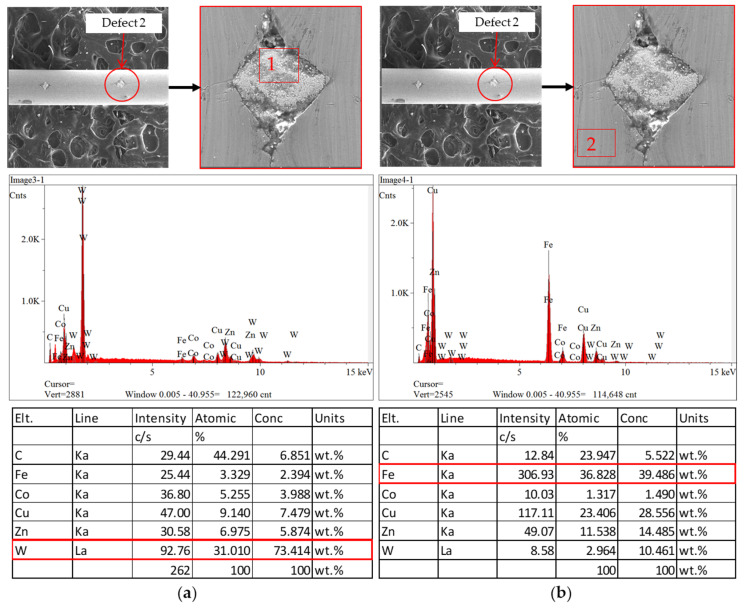
Results of EDS analysis for inclusion defect 2. (**a**) Inside of defect; (**b**) outside of defect.

In order to compare the different materials inside and outside the inclusion defects, EDS analysis was performed on both regions, and it was repeated for the second defect on the same sample. As illustrated in Figure 12a, tungsten carbide is the main material inside the defect, while iron (Fe) is the main ingredient outside of the defect as shown in Figure 12b. The EDS results show that this defect was caused by the external source from which the tungsten carbide originated. To verify the abnormal material adhered to the inclusion defect, EDS was performed again on the second defect, and the same result was obtained when tungsten carbide was found to be the main factor inside the defect, as shown in Figure 13a, while no abnormal material was found on the outside of the defect, as illustrated in Figure 13b.

Considering the fact that tungsten carbide is utilized to produce dies for the drawing process, it is likely for the broken pieces of dies to become stuck inside the steel wire during the drawing process due to the high hardness of tungsten carbide compared to that of steel. Additionally, the drawing process takes place under high pressure and in a single direction, making it easier for the broken die pieces to insert into the surface of the steel filament. Therefore, the primary reason for the inclusion defect containing tungsten carbide is likely due to the broken dies during the drawing processes.

For the scratch defects, there is no room for doubt that the micro surface defects are the scratches including the start and end points. It is important to narrow down which process caused these scratches. Furthermore, since the steel wire was coated with a brass layer before going to the wet drawing process, an investigation was conducted to determine whether the scratched defects were created during wet-drawing or by the previous processes. The brass-coating layer was also analyzed using EDS.

**Figure 14:**
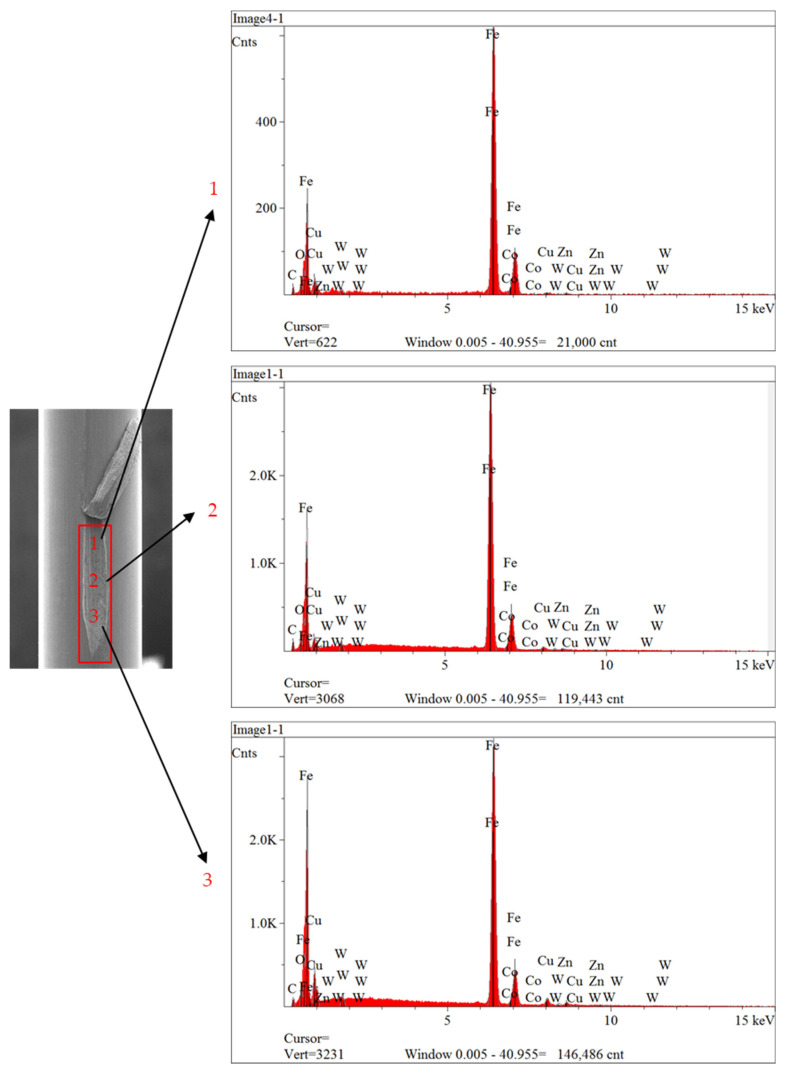
Results of EDS analysis inside of scratch defect.

**Figure 15:**
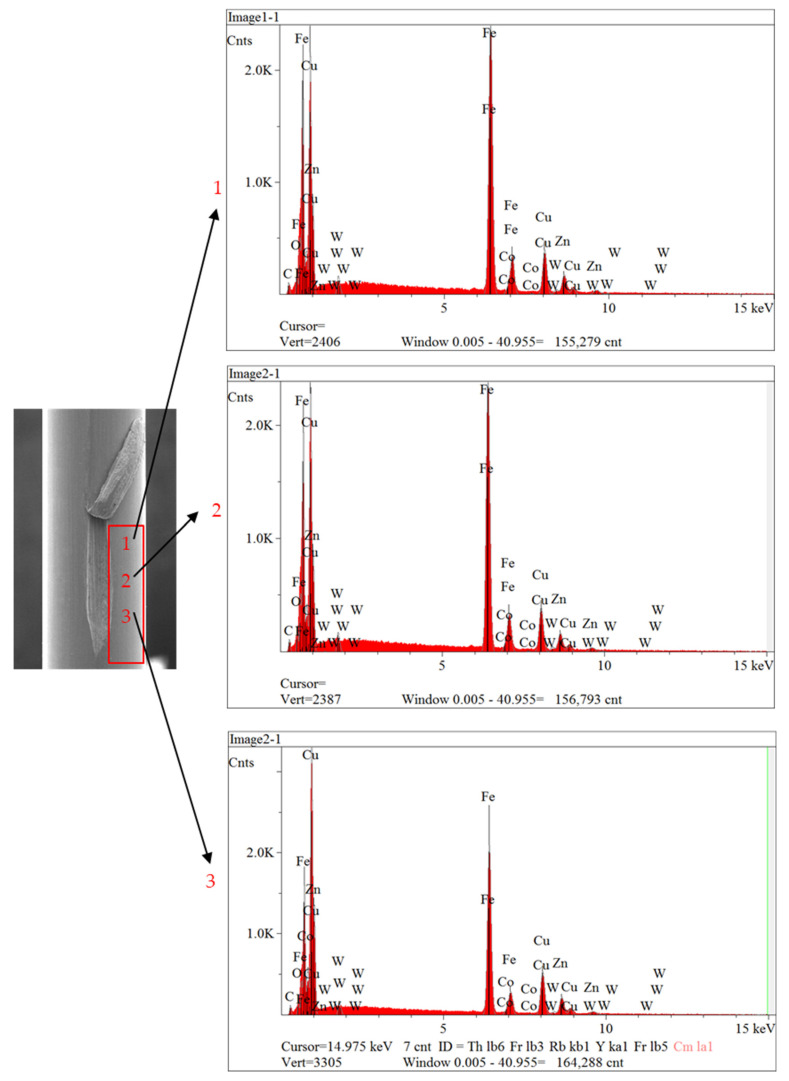
Results of EDS analysis outside of scratch defect.

The analysis was conducted two times at three places (1, 2 and 3) inside and outside (normal region) the scratch defect, as shown in Figure 14 and Figure 15. It can be seen from results of the analysis that brass was absent from all regions inside the scratch, as illustrated in Figure 14. However, brass was still present on the surfaces of the normal area, as indicated in Figure 15. Therefore, these surface scratches were caused during the wet-drawing process.

## 5. Conclusions

In this paper, a comprehensive methodology was introduced for detecting, verifying and analyzing micro surface defects in steel filament, using Eddy current principle and EDS technique. The sensor coil was utilized with created defects to establish the working conditions and to separate unacceptable products with surface defects. 

The defect rate stood at 26.37% out of a total of 182 produced samples. The detected surface defects appeared randomly along the length of the product and were also verified manually by pulling back the wire and comparing these results to those obtained from the sensor system during the experiment, which confirmed the accuracy of this methodology to detect the surface defects. Two common surface defects have been identified: the first is a longitudinal scratch defect characterized by both a start and an end point, while the second defect exbibits a diamond shape and contains an external material referred to as an inclusion. Furthermore, EDS analysis confirmed that the root cause of the inclusion defect was tungsten carbide, which is the unique material used for drawing dies. The second defect was also confirmed to have been caused during the wet-drawing process owing to the difference in the brass-coating layer between inside and outside of the defects.

This innovative approach demonstrates the capability to precisely identify surface defects on 0.5 mm steel filaments at an exceptional speed of 400 m per minute. By accurately pinpointing the location of these defects, the methodology facilitates in-depth analysis essential for quality control and root-cause investigations. This pioneering contribution not only advances the efficiency of defect identification and analysis but also sets a new standard for quality management in high-speed steel-filament-production processes, signifying a valuable step towards improved manufacturing efficiency and product quality assurance.

## Figures and Tables

**Table 1:** Artificial crack dimensions.

Crack No.	Depth	Width
1	20	73
2	80	73

**Table 2:** Summary of experimental results.

Categories	Number of Samples	Pass/Fail
Without defect	134	Pass
With defect	48	Fail
Total samples	182	
Defective ratio	26.37%	

## Data Availability

Not applicable.

## References

[1] Yilmaz M., Ertunc H.M. (2007). The prediction of mechanical behavior for steel wires and cord materials using neural networks. Mater. Des..

[2] Lee S.W., Jeong K.M., Kim K.W., Kim J.H. (2018). Numerical estimation of the uneven wear of passenger car tires. World J. Eng. Technol..

[3] Polyakova M., Stolyarov A. (2021). Automobile Tires’ High-Carbon Steel Wire. Encyclopedia.

[4] Wu B., Wang Y.J., Liu X.C., He C.F. (2015). A novel TMR-based MFL sensor for steel wire rope inspection using the orthogonal test method. Smart Mater. Struct..

[5] Tran K.S. Detection of Micro-scratch Found on Surface of Steel Filament Using Eddy Current Sensor. Proceedings of the First Australian International Conference on Industrial Engineering and Operations Management.

[6] Zapata J., Vilar R., Ruiz R. (2011). Performance evaluation of an automatic inspection system of weld defects in radiographic images based on neuro-classifiers. Expert Syst. Appl..

[7] Shirinzadeh B., Alici G., Foong C.W., Cassidy G. (2004). Fabrication process of open surfaces by robotic fibre placement. Robot. Comput. Integr. Manuf..

[8] Batty T., Ehrampoosh A., Shirinzadeh B., Zhong Y., Smith J. (2022). A transparent teleoperated robotic surgical system with predictive haptic feedback and force modelling. Sensors.

[9] García-Martín J., Gómez-Gil J., Vázquez-Sánchez E. (2011). Non-destructive techniques based on eddy current testing. Sensors.

[10] Zhao P., Shirinzadeh B., He X., Guo J., Shi K., Qiang B., Jin Q., Li F. (2022). Predicting and Improving Interlaminar Bonding Uniformity during the Robotic Fiber Steering Process. Polymers.

[11] Zhang G., Tang Z., Fan Y., Liu J., Jahanshahi H., Aly A.A. (2021). Steel Wire Rope Surface Defect Detection Based on Segmentation Template and Spatiotemporal Gray Sample Set. Sensors.

[12] Su S., Ma X., Wang W., Yang Y. (2019). Stress-Dependent Magnetic Charge Model for Micro-Defects of Steel Wire Based on the Magnetic Memory Method. Res. Nondestruct. Eval..

[13] Shirinzadeh B., Teoh P.L., Tian Y., Dalvand M.M., Zhong Y., Liaw H.C. (2010). Laser interferometry-based guidance methodology for high precision positioning of mechanisms and robots. Robot. Comput. Integr. Manuf..

[14] Ammar M.M., Shirinzadeh B. (2022). Evaluation of robotic fiber placement effect on process-induced residual stresses using incremental hole-drilling method. Polym. Compos..

[15] Saludes-Rodil S., Baeyens E., Rodriguez-Juan C.P. (2015). Unsupervised classification of surface defects in wire rod production obtained by eddy current sensors. Sensors.

[16] Yoshida K., Norasethasopon S., Shinohara T., Ido R. (2003). Influence of flaws of wire rod surface, inclusions and voids on wire breaks in superfine wire drawing. JSME Int. J. Ser. A Solid Mech. Mater. Eng..

[17] Verpoest I., Aernoudt E., Deruyttere A., De Bondt M. (1985). The fatigue threshold, surface condition and fatigue limit of steel wire. Int. J. Fatigue.

[18] Tran K., Phan H., Lee H., Kim Y., Park H. (2016). Blocking force of a piezoelectric stack actuator made of single crystal layers (PMN-29PT). Smart Mater. Struct..

[19] Tran K., Lee H., Kim Y., Park H. (2016). Resonant frequency and hysteresis of a stack actuator made of single crystal (PMN-29PT) layers. Smart Mater. Struct..

[20] Ammar M.M.A., Shirinzadeh B., Zhao P., Shi Y. (2021). An approach for damage initiation and propagation in metal and carbon fiber hybrid composites manufactured by robotic fiber placement. Compos. Struct..

[21] Shinohara T., Yoshida K. (2005). Deformation analysis of surface flaws in stainless steel wire drawing. J. Mater. Process. Technol..

[22] Yun J.P., Choi D.-C., Jeon Y.-J., Park C., Kim S.W. (2013). Defect inspection system for steel wire rods produced by hot rolling process. Int. J. Adv. Manuf. Technol..

[23] Zhou P., Zhou G., Wang S., Wang H., He Z., Yan X. (2022). Visual Sensing Inspection for the Surface Damage of Steel Wire Ropes with Object Detection Method. IEEE Sens. J..

[24] Liu Q., Tian Y., Zhai J., Tian L., Chen L., Chen L. (2020). Prediction of surface wrinkle defect of welding wire steel ER70S-6 in hot bar rolling process using finite element method and experiments. Metals.

[25] Heo T., Cho S.W., Cho S.H., Ahn B., Lim Z.S. (2017). Detection of an axial surface microcrack in steel wire rods with noncontact ultrasonic reverberation spectroscopy. J. Mech. Sci. Technol..

[26] Jasper W.J., Potlapalli H. (1995). Image analysis of mispicks in woven fabric. Text. Res. J..

[27] Conci A., Proença C. (2000). A computer vision approach for textile inspection. Text. Res. J..

[28] Lane J.S. (1998). Textile Fabric Inspection System. U.S. Patent.

[29] Daugman J.G. (1985). Uncertainty relation for resolution in space, spatial frequency, and orientation optimized by two-dimensional visual cortical filters. JOSA A.

[30] Sari-Sarraf H., Goddard J.S. Vision system for on-loom fabric inspection. Proceedings of the 1998 IEEE Annual Textile, Fiber and Film Industry Technical Conference (Cat. No. 98CH36246).

[31] Filipovic M. (2007). Evolution of Artificial Defects during Shape Rolling. Doctoral Dissertation.

[32] Yun J.P., Choi S., Kim S.W. (2009). Vision-based defect detection of scale-covered steel billet surfaces. Opt. Eng..

[33] Park C., Choi S., Won S. (2010). Vision-based inspection for periodic defects in steel wire rod production. Opt. Eng..

[34] Zhang W., Bu J., Li D., Zhang K., Zhou M. (2022). Coupling Interference between Eddy Current Sensors for the Radial Displacement Measurement of a Cylindrical Target. Sensors.

[35] Ramos H.G., Postolache O., Alegria F.C., Ribeiro A.L. Using the skin effect to estimate cracks depths in mettalic structures. Proceedings of the 2009 IEEE Instrumentation and Measurement Technology Conference.

[36] Wikipedia Electrical Resistivity and Conductivity. https://en.wikipedia.org/wiki/Electrical_resistivity_and_conductivity.

[37] Wikipedia Permeability (Electromagnetism). https://en.wikipedia.org/wiki/Permeability_(electromagnetism).

